# Special Issue “New Insights into Bioactive Peptides: Design, Synthesis, Structure–Activity Relationship (2nd Edition)”

**DOI:** 10.3390/ijms27177587

**Published:** 2026-08-25

**Authors:** Flavia Anna Mercurio, Marilisa Leone

**Affiliations:** Institute of Biostructures and Bioimaging (IBB), National Research Council of Italy (CNR), Via Pietro Castellino 111, 80131 Naples, Italy

Peptides are molecules characterized by sequences of amino acids linked through peptide bonds. They are practically proteins provided with shorter amino acid chains. Human endogenous peptides are produced in cells, while exogenous peptides can originate from other living organisms and can be introduced into the body, for example, through foods and medications [1]. Exogenous peptides can also be obtained in laboratories by synthetic routes or engineered cells, as well as with de novo designed sequences with respect to natural peptides [1]. Endogenous peptides include hormones and neurotransmitters that are fundamental in modulating certain physiological activities [2]. Studies on endogenous peptides marked the beginning of the research field on peptide therapeutics and led in the last century to the production of synthetic insulin as a drug for diabetes treatment [2]. Thereafter, much progress has been made in peptide drug development, due to advancements in synthetic and analytic technologies, as well as in structural biology and in silico tools, including new-generation cutting-edge ones relying on artificial intelligence (AI), to support drug discovery [2,3]. While pharmaceutical companies initially looked exclusively to small molecules to develop original therapeutics, bioactive peptides have recently emerged as new efficacious drug modalities.

In fact, peptide therapeutics stand halfway between the other two classes of molecules generally employed as drugs (i.e., small molecules and biological macromolecules like proteins/antibodies) and preserve a few of the advantages of both classes of compounds. For example, similarly to proteins and antibodies, peptide therapeutics might possess high target specificity due to their larger dimensions, bringing less side-effects. But at the same time, they might be characterized by lower immunogenicity and production costs than the other macromolecule therapeutics as well [2,4]. Moreover, with respect to small molecules used as drugs, they are more suitable for targeting disease-related proteins or protein–protein interactions (PPIs). Nevertheless, compared with small molecules, peptides show increased flexibility that, along with their increased sizes, allow them to better occupy the large-interface areas characterizing PPIs [2,3]. Flexible peptides generally lack a well-folded structure organization, and the absence of secondary structure motives often causes a decrease in binding affinity towards the target proteins. This limitation inspired synthetic chemistry strategies to made canonical peptide sequences more rigid, such as peptide cyclization [5]. Other drawbacks of peptide therapeutics include poor cell permeability and plasma stability. Cell permeability can be overcome through methods improving peptide cellular uptake, including the design of peptide sequences that are able to cross the membrane bilayer, known as cell-penetrating peptides (CPPs), which can be joined to peptide chains of interest [6]. On the other end, peptide stability and resistance to proteolytic degradation can be enhanced by peptide sequence modifications through, for example, the insertion of non-proteinogenic amino acids or cyclization [2,3].

Natural proteins represent a relevant source of short peptide sequences to be screened in drug discovery studies; peptide libraries can be extrapolated through different techniques, including enzymatic hydrolysis [7]. These exogenous peptides can be retrieved from proteins of any kind of organism, mainly from animals, plants, or microbes, and find a large-scale field of application.

Natural peptides attract attention as therapeutic compounds able to modulate a wide range of pathologies, including infectious diseases, inflammations, and cancer [7]. Certain peptide properties, like the tuneable sequences, are also attractive for the development of biomaterials for biomedical applications [8]. For example, peptide–drug conjugates have emerged as useful tools for drug delivery; meanwhile, peptides that can self-assemble into a variety of nanostructures can also be implemented, among all possible applications, as biosensors or drug release tools [8].

This Special Issue, which concerns different aspects covered by the research field of bioactive peptides, is in its second edition, and includes eight research articles and two reviews.

The communication by Li et al. [9] is related to peptides with antimicrobial and anticancer potential working as inhibitors of the SPSB2 (SPRY (SPla and RYanodine receptor) domain-containing SOCS (Suppressor of Cytokine Signaling) box protein 2) protein. SPSB2 belongs to a family of proteins containing a SPRY/B30.2 domain known to bind the N-terminal region of iNOS (Inducible Nitric Oxide Synthase) and induce its proteasomal degradation by recruiting an E3 ubiquitin ligase complex responsible for iNOS polyubiquitination. Inhibition of the interaction between SPSB proteins and iNOS could induce an increase in cellular NO levels with consequent antimicrobial and anticancer outcomes. The cyclic peptide cR8 was first identified as a weak SPSB2 ligand. Two peptide analogs with improved binding affinity for SPSB2 with respect to cR8 were next generated (i.e., “cR7” and “cR9”). The design of these new peptides was driven by a structure-based approach, and details characterizing the SPSB2/cR7 and/cR9 complexes were unveiled by X-Ray crystallography. Displacement experiments were also carried out to investigate cR7’s and cR9’s ability to push iNOS away from binding to diverse SPSB proteins in macrophage cell lysates. The cR7 peptide was finally identified as the strongest inhibitor of iNOS binding to SPSB2, SPSB1, and SPSB4 proteins [9]. The results of this work are a clever example of structure-based peptide optimization, which adds information useful in guiding their optimization in terms of specificity to single SPSB proteins.

Studies from our laboratory embrace the computational drug design of peptides with anticancer potential [10]. In this case, the target cancer-related PPI is represented by the interface between the Sam (Sterile alpha motif) domain of the lipid phosphatase Ship2 (Ship2-Sam) and the Sam domain of the receptor tyrosine kinase EphA2 (EphA2-Sam), which plays a pivotal, well-established role in cancer. Sam domains are small five-helix bundle protein binding modules; potential peptide inhibitors of the EphA2-Sam/Ship2-Sam complex were designed by a computational strategy including the use of the FoldX (v. 5) [11] software. A virtual library of peptides was first generated by modifying the sequence of a previously found peptide ligand of Ship2-Sam, after which a few mutant sequences were selected to be experimentally validated in terms of binding propensities to Ship2-Sam by NMR (Nuclear Magnetic Resonance) and BLI (BioLayer Interferometry) experiments. Meanwhile, cell-based assays were implemented to assess the peptide cytotoxic effects and their possible influence on the EphA2 pathway. Our work proposes a strategy to optimize, by a rational in silico-based approach, peptide sequences to be tested as inhibitors of the EphA2-Sam/Ship2-Sam complex, but it could be applied to different PPIs involved in a variety of pathologies [10]. Our work also pointed out that the workflow adopted for the computational design of peptide inhibitors needs to be carefully adjusted by iteratively changing several key software parameters to heighten the chance of obtaining peptide libraries with real abilities to interfere with PPIs.

The manuscript of Golonka et al. is related to cationic amphipathic peptides with anticancer potential [12]. This class of peptides, derived from antimicrobial peptides or de novo designed, have a potential anticancer effect as their sequences, rich in positively charged residues, give them better capacity to interact with the membrane of cancer cells characterized by an increased level of negative charges, while the hydrophobic/amphipathic character favors interaction with the lipidic membrane bilayer. Amphipathic peptides may thus damage cancer cells due to membrane destabilization, or may result in penetration inside the cell and the induction of apoptosis. In this study, several peptides with different numbers of lysine and tryptophan residues, or modified with a C12 alkyl chain and thus with various amphipathic characteristics, were analyzed for their ability to either interact or destabilize cancer cell membranes through several techniques. The physiochemical properties of the peptides were analyzed, along with their cytotoxic effects in melanoma and normal cell lines and their ability to bind POPG (phosphatidylglycerol) model membranes. The effects of peptides on cytoskeleton organization and membrane permeability were also assessed. The main results of this work emphasized that a proper balance between charge and hydrophobicity within a specific sequence arrangement needs to be considered for the design of selective anticancer peptides [12].

Two research articles of this Special Issue focused on the discovery of natural peptides derived from the proteolytic digestion of proteins from different natural sources. In one of these articles, bioinformatic tools were entirely implemented to evaluate the inhibitory potential of peptides from Lupin (*Lupinus angustifolius* L.) seeds towards DPP-IV (Dipeptidyl peptidase IV) and ACE-I (Angiotensin I-Converting Enzyme) activities to treat diabetic patients with hypertension [13]. Indeed, inhibition of DPP-IV is desirable to increase the levels of hormones suitable for controlling plasma glucose levels, such as glucose-dependent insulinotropic polypeptide and glucagon-like peptide 1, while ACE-I blockage avoids the formation of angiotensin II, which is a vasoconstrictor, and the cleavage of bradykinin, which is a vasodilator. The BIOPEP (BIOactive PEPtides)-UWM platform [14] was employed for in silico hydrolysis of Lupin proteins to predict bioactive peptides derived by the action of different enzymes such as ficin, bromelain, and papain. The ADMETlab 2.0 (Absorption, Distribution, Metabolism, Excretion, and Toxicity lab 2.0) tool [15] was used to predict the pharmacokinetic properties of the identified peptides, and docking studies through Autodock Vina (v. 1.1.2.) [16,17] were also conducted to filter out peptides with potential higher binding affinity to target proteins. Taken together, these computational tools predicted two peptides provided with good ADMET profiles, i.e., Glu-Tyr and Ile-Ala-Tyr, as possible strong ligands of DPP-IV and ACE-I, respectively. These studies suggested an in silico-based strategy for the selection of the most interesting bioactive peptides, which of course can only be a starting point for further in vitro and in vivo experiments to assess their real therapeutic power [13].

In the contribution by Lin et al. [18], the hydrolysates from gliadin, whey, and casein proteins obtained using bromelain, papain, and thermolysin proteolytic digestion were evaluated as inhibitors of BACE1 (β-site Amyloid precursor protein Cleaving Enzyme 1), a target for Alzheimer’s disease (AD) treatment involved in amyloid-β (Aβ) peptide production in the brain. In vitro experiments identified bromelain-hydrolyzed gliadin (G-Bro) as the best BACE1 inhibitor. BACE1 expression and the extracellular levels of APP (Amyloid Precursor Protein), moreover, were reduced in a murine neuroblastoma cell line treated with G-Bro and, therefore, Aβ fiber formation was inhibited. Considering the sequences of the peptides comprising the G-Bro, identified by mass spectrometry, the authors hypothesized a mechanism of Aβ aggregation inhibition involving proline residues as β-sheets breakers, lysine residues that interfere with aggregation by introducing positive charges interacting with the Aβ-negative ones, hydrophobic interactions stabilizing peptide binding, and glutamine residues that could enhance peptide solubility and stability. These features related to peptide sequences must of course be validated to clarify their impact on the interaction with Aβ, as well as the therapeutic effects of peptides in animal models, if they need to lay the foundation for future studies of natural BACE1 inhibitors for AD prevention [18].

Another manuscript included in this Special Issue investigated peptide therapeutics against AD [19]. Litus et al. explored the mechanisms by which glucagon-like peptide 1 receptor agonists (GLP-1RAs), used for diabetes treatments, can influence Aβ metabolism, thus functioning as therapeutics for AD. The tested GLP-1RAs consisted of an N-terminal truncated glucagon-like peptide 1 (GLP-1(7-37)) and its analogs, semaglutide (Sema), liraglutide (Lira), and exenatide (Exen). The ability of GLP-1Ras to bind the monomeric forms of Aβ40 and Aβ42 was investigated, and the largest binding affinity was evaluated for Lira. DLS (Dynamic Light Scattering) experiments were used to investigate the quaternary structure of GLP-1RAs, which resulted in all being aggregation-prone, a feature that could influence the binding to Aβ. The effects of GLP-1RAs on Aβ fibrillation and the influence of peptides in affecting Aβ40- or Aβ42-induced cytotoxicity were also investigated and revealed contrasting behaviors among them. Thus, the effects of the tested peptides on Aβ40 fibrillation and Aβ-induced cytotoxicity resulted in them being dependent on the specific GLP-1RA and potentially correlating with a diverse ability to cross the blood–brain barrier. Although the role of GLP-1RAs on Aβ metabolism must be further assessed in in vivo studies and many efforts need to be devoted to better understand their mechanisms of action and structure–activity relationship, the results of this work encourage the development of GLP-1RA analogs, leading to more pronounced effects as anti-AD therapeutics [19].

Nonribosomal peptides (NRPs) are a class of natural peptides characterized by different structures and a variety of functions, acting for example as antibiotics, immunosuppressants, or anticancer agents [20]. These kinds of peptides are synthesized by the nonribosomal peptide synthetase machinery that contains a Condensation (C) domain responsible for amide bond formation during peptide elongation. Due to the challenges faced in obtaining NRPs through synthetic routes, the engineering of the nonribosomal peptide synthetase is needed to produce them. However, this approach might cause reduced yields compared to those achieved with the natural enzyme, thus suggesting that more should be understood about its biosynthetic mechanism, particularly concerning the C domain. The contribution of Huang et al. [20] consisted of a review specifically focused on different features of the C domain, such as its structure, the mechanism of action, and state-of-the-art engineering approaches. Controversial aspects of the C domain related to the catalytic mechanism and the substrate selectivity are deeply discussed, as they are not yet fully clarified [20].

In the manuscript of Gitlin-Domagalska et al., which focused on techniques to obtain peptides with enhanced oral bioavailability, oxytocin (OT) prodrugs derived by the Lipophilic Prodrug Charge Masking (LPCM) strategy were investigated [21]. OT is a peptide neurohormone produced in the hypothalamus that can be used to increase social behaviors in autistic individuals affected by dysregulated OT levels, but it is characterized by poor oral bioavailability. LPCM is a technique used to produce prodrugs with facilitated transport across the intestinal epithelium by modulating the hydrophilic peptide charges with the addition of alkoxycarbonyl groups, cleaved by esterases only after peptide intestinal absorption. Several OT prodrugs were thus synthesized by modifying the peptide N-terminal amino group with alkoxycarbonyl groups composed of alkyl chains of different length, and their membrane permeability was assessed through PAMPA (Parallel Artificial Membrane Permeability Assay) and in-cell experiments. These studies provided evidence that different OT prodrugs were characterized by possibly improved oral availability compared to the unmodified OT peptide, and also suggested that the length of the alkyl chain used to generate the peptide prodrugs should be fine-tuned, as derivatives with higher hydrophobicity showed low permeability probably due to strong interactions with the membrane. The results of this study were promising and paved the way for optimization of the LPCM strategy to improve peptide oral bioavailability [21].

The communication of Soriano et al. [22] explored peptide-based biomaterials for biomedical applications by developing a microfluidic-based approach including tripeptide hydrogels as sorbents for extraction of drugs from saliva. A microfiber was developed starting from the tripeptide DLeu-LPhe-LPhe through microfluidic-directed assembly. The conformation of peptide fibers was analyzed, providing evidence of the formation of β-sheet assemblies, which were not modified after incorporation of small molecules. The peptide fibers were shown to successfully co-assemble with the drugs 5-fluorouracil (5-FU) and naproxen and to have a relevant drug-adsorption ability in saliva samples. The designed tripeptide hydrogel is a promising sorbent material that can be used for rapid detection of drug levels in oral fluids and thus can be applied to the supervision of therapeutic drug levels and for toxicological analyses [22].

The review of Fehérvári et al. [23] approached the field of drug delivery through peptide–drug conjugates (PDCs), which attract large attention in targeted anticancer therapies as a route to deliver chemotherapeutic or cytotoxic drugs with fewer side-effects than standard therapies. In particular, anthracycline-containing peptide conjugates are very promising in delivering cytotoxic drugs to cancer cells, but of course, these PDCs need to be properly designed and analyzed during drug development projects to evaluate their mechanism of action and safety. The major challenges encountered in anthracycline-containing peptide conjugate development are related to their analytical characterization through mass spectrometry (MS) due to the structural complexity of these molecules. The review is thus focused on discussing the technical issues related to the MS analysis of these PDCs [23].

In conclusion, the second edition of this Special Issue again reflects the variety of biomedical applications peptides can be involved in, as well as the continuous efforts the research community is devoted in finding novel peptide molecules as therapeutics. Indeed, most of the manuscripts published here are related to the design and optimization of peptides targeting PPIs involved in diseases, including cancer and AD [9,10,18,19], or to obtain peptides able to penetrate or destabilize cancer cell membranes with cytotoxic potential [12]. In this context, in two manuscripts, the therapeutic potential of peptides derived from enzymatic digestion of naturally occurring proteins is explored [13,18]. Another aspect that emerges is the implementation of different computational tools flanking the discovery of novel peptide therapeutics, reflecting the advancements of in silico approaches to support the design of experimental procedures or to speed up the selection of peptides to be submitted to experimental validation among members of very large virtual peptide libraries [10,13]. Strategies to overcome peptide drawbacks related to poor in vivo stabilities are also discussed, and a possible approach to generate peptide prodrugs with increased oral bioavailability is proposed [21]. Besides the employment of peptides as therapeutics, different technical aspects related to the design of efficacious drug delivery tools featuring drug–peptide conjugates [23] or to the engineering of the catalytic domain of the nonribosomal peptide synthetase to produce peptides in high yields [20] are other appealing topics of this Special Issue. Finally, an interesting example of a biomaterial constituted by a peptide hydrogel-based sorbent, which could be implemented to retrieve drugs from saliva, is included within this Special Issue [22]. From the collected articles also emerges the need for research attempts to overcome some limitations bioactive peptides still suffer in terms of production, evaluation through analytical tools, not optimal binding affinity to target PPI, and applicability as therapeutics. Moreover, although in silico tools available to virtual peptide screening and structure prediction of peptide-based complexes are rapidly evolving, thanks to the large recent employment of AI tools, and are increasingly focused on this class of molecules, these instruments produce outcomes that still need to be validated by expensive and time-consuming experimental work. Due to the growing relevance of bioactive peptides as therapeutics, further innovations are expected in the near future to overcome all cited drawbacks related to this research field, and we can envision that a great step forward will be provided by the development of original AI applications. In the end, given the vast array of topics, the cutting-edge techniques, and the implications for human health of the peptide science that cover the works collected within this second edition of the peptide-based Special Issue, we believe it will be a source of inspiration for other scientists from both Academia and Pharma working in the drug discovery field.
